# *Taccakhanhhoaensis* V.S. Dang & Vuong (Taccaceae), a new species from southern Vietnam

**DOI:** 10.3897/phytokeys.114.29367

**Published:** 2018-12-31

**Authors:** Van Son Dang, Ba Vuong Truong, Thi Phuong Thao Nguyen, Nghia Son Hoang

**Affiliations:** 1 Institute of Tropical Biology, Vietnam Academy of Science and Technology, 85 Tran Quoc Toan Street, District 3, Ho Chi Minh City, Vietnam Institute of Tropical Biology, Vietnam Academy of Science and Technology Ho Chi Minh Vietnam

**Keywords:** Hon Ba Nature Reserve, Khanh Hoa, Taccaceae, Taxonomy, Vietnam

## Abstract

*Taccakhanhhoaensis* V.S. Dang & Vuong (Taccaceae) is described as a new species from Hon Ba Nature Reserve in southern Vietnam. This species is morphologically similar to *T.chantrieri* and *T.ampliplacenta* but differs from its allies by several salient characters: size of leaves and petioles, inflorescent much shorter leaves, number of flowers, stigma lobes, buds colour. A description, conservation assessment, together with photographs and a key to the species of *Tacca* in Vietnam are presented.

## Introduction

The family Taccaceae is composed of only one genus, *Tacca* J.R. Forster & G. Forster and its 11 species (Ding et al. 2000). Its species are distributed mainly in tropical regions of Asia and Oceania, except for *T.parkeri* Seem. known only in South America (Ding and Larsen 2000, Drenth 1972, 1976). Currently, six species of the genus *Tacca* have been recorded in Vietnam: *T.chantrieri* André, *T.integrifolia* Ker Gawl., *T.leontopetaloides* (L.) Kuntze, *T.palmate* Blume, *T.plantaginea* (Hance) Drenth and *T.subflabellata* P.P. Ling & C.Ting (Pham 2000, Vo 2007).

During botanical field surveys in Hon Ba Nature Reserve, Khanh Hoa Province, southern Vietnam in 2017, a species of *Tacca* was collected. After thorough examination of the *Tacca* species in Drenth (1972, 1976), Ding and Larsen (2000), Pham (2000), Vo (2007) and Zhang et al. (2005, 2006), re-examination of specimens deposited in the Vietnamese herbaria VNM, HN and VNMN, as well as specimen images on the website of JSTOR Global Plants, P and K, we concluded that our sample belongs to a new species, which we describe in this study.

## Materials and methods

**Figure 1.**
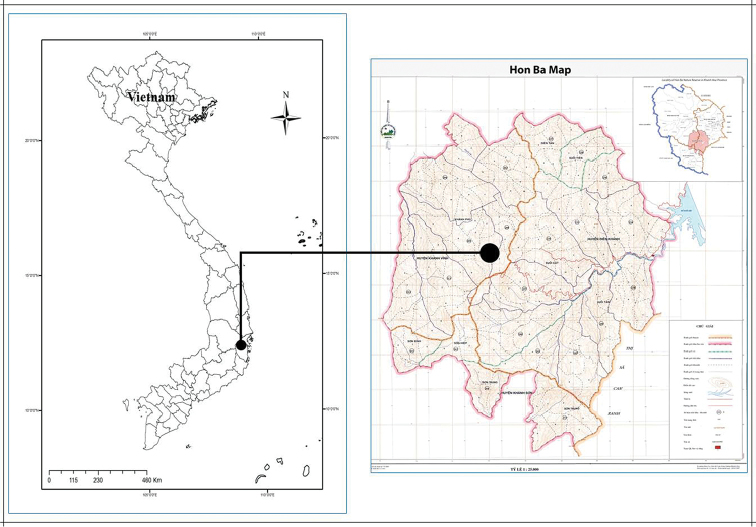
Type locality of *Taccakhanhhoaensis* V.S. Dang & Vuong.

The description of the new species was based on material collected in Hon Ba Nature Reserve (over 19,000 ha), Khanh Hoa Province, southern Vietnam (Figure 1). The measurements and description were prepared from living plants with a ruler accurate to 0.5 mm. Herbarium material was dried and preserved in 70% ethanol and stored at the Institute of Tropical Biology (VNM). All the photos were taken with a Canon 600D fitted with a EF–S 60 mm f/2.8 Macro USM lens.

## Taxonomy

### Tacca khanhhoaensis

Taxon classificationPlantaeDioscorealesTaccaceae

V.S. Dang & Vuong sp. nov.

urn:lsid:ipni.org:names:77192866-1

Figures 2 , 3

#### Diagnosis.

*Taccakhanhhoaensis* is similar to *Taccachantrieri* André in leaf shape and bracts, but differs from it in having shorter leaves (up to 45 cm vs. 60 cm long) and petioles (up to 22 cm vs. 43 cm long), inflorescences with fewer flowers (5 to 10 vs. 15 to 25), 2-lobed stigma vs. 3-lobed stigma and dark red buds vs. green buds. The new species is also somewhat similar to *T.ampliplacenta* L. Zhang & Q.-J. Li of China, but differs from it by having smaller leaves (30–45 × 10–14 cm vs. 55 cm long) and fruits (3–4 × 1.5–2 cm vs. 4–6 × 2–2.5 cm) and very short petioles (22 cm vs. 50 cm long), inflorescent much shorter leaves (vs. longer), number of flowers (5 to 10 vs. 25) and dark red buds vs. black-purple.

**Figure 2.**
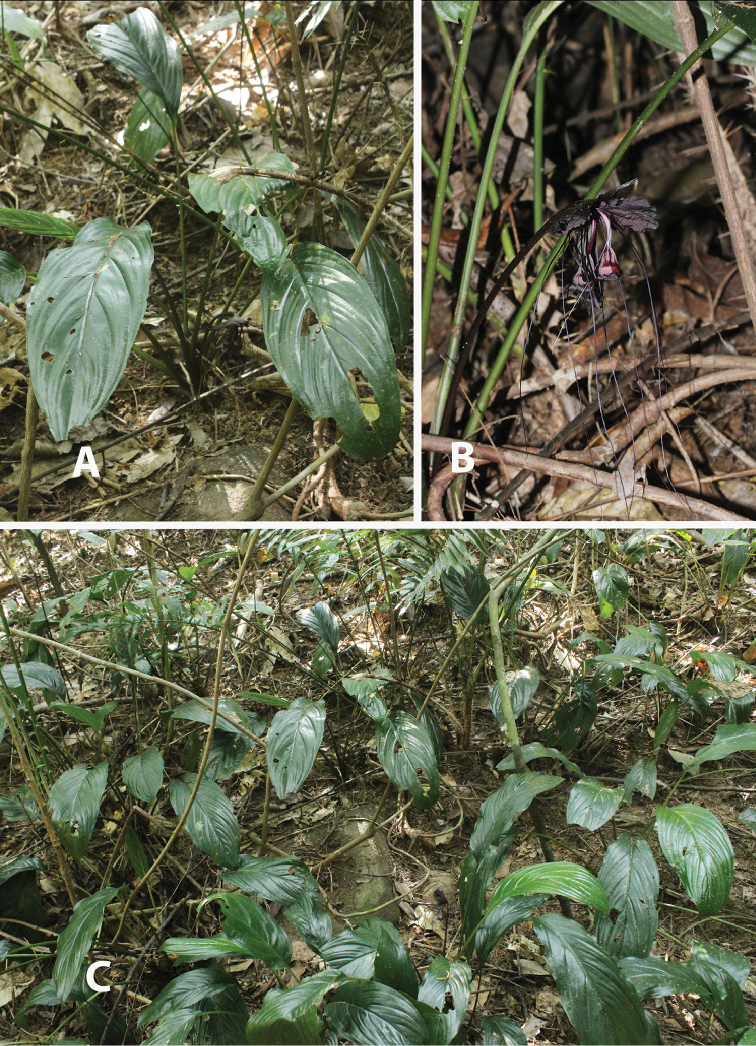
*Taccakhanhhoaensis* V.S. Dang & Vuong. **A** Habit **B** Inflorescent much shorter leaves **C** Habitat.

**Figure 3.**
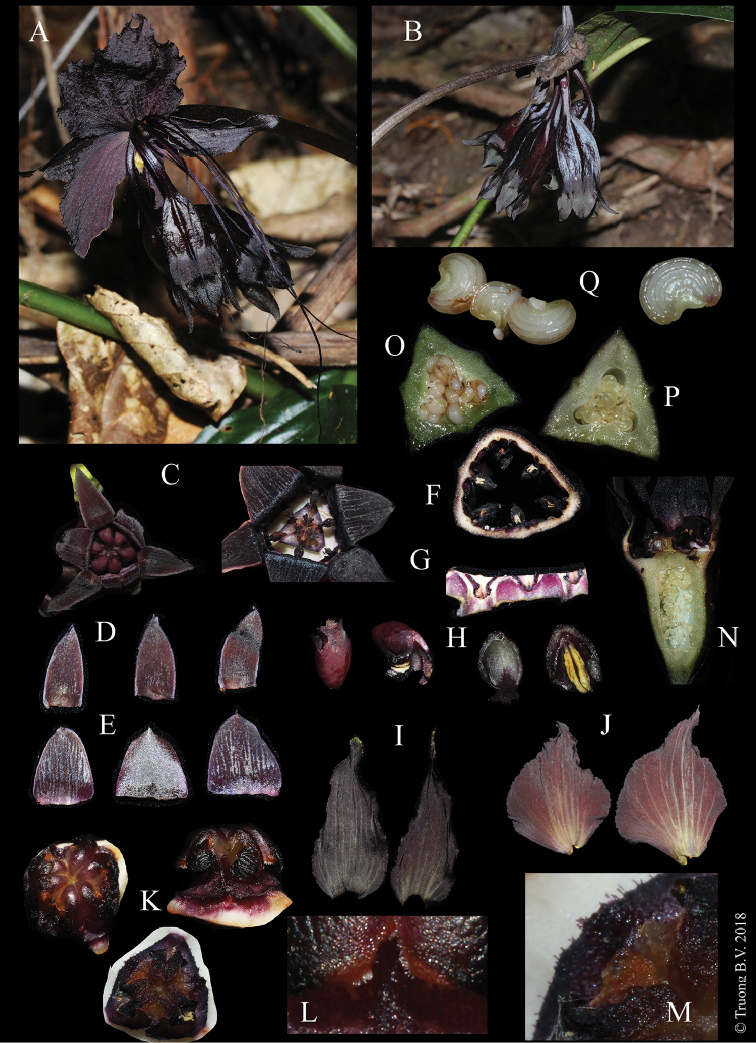
*Taccakhanhhoaensis* V.S. Dang & Vuong. **A** Inflorescence **B** Fruits **C** Flowers, top view showing perianth lobes, stamens and stigma **D** Outer perianth lobes **E** Inner perianth lobes **F** Inner surface of perianth tube **G** Anther and connective **H** Longitudinal section of Anther with a pair of theca **I** Outer involucral bracts **J** Inner involucral bracts **K** Stigmas **L** Finely pubescent hairs at apex of stigma lobes **M** Black hairs on style **N** Longitudinal section of flower **O** Transverse section of fruit **P** Transverse section of ovary **Q** Seeds.

#### Type.

VIETNAM. Khanh Hoa Province, Hon Ba Nature Reserve, terrestrial under the shade of the canopy on dry soil or close to the stream, alt. 353 m, 12°06.36'N, 108°59.46'E, 16 April 2017, *Truong Ba Vuong, Mang Van Lam, Dang Van Son, BV256* (holotype VNM!; isotype VNM!, the herbarium of Hon Ba Nature Reserve!).

#### Description.

Perennial herbs. *Rhizome* cylindric, fleshy, with many stout roots. *Leaves* radical, 5 to 8 leaves; laminae oblong to obovate or oblong-elliptic, 30–45 × 10–14 cm, apex acute, base attenuate, margin slightly undulate; main veins pinnate; petiole erect, 14–22 × 0.5–1 cm, green to purplish-green, sheathing at base. *Inflorescence* umbel, 5–10-flowered; scape erect, 20–38 cm long, nodding, pale green-purple at base, more dark purple above. *Involucral bracts* 4, decussate, sessile; two outer bracts vertical arrangement, unequally, ovate-lanceolate, 4.5–5.5 × 2–2.5 cm, black-purple, apex acuminate, base obtuse; two inner bracts horizontal arrangement, broadly ovate, 6–6.5 × 4–5 cm, deep purple red with dark purple at margin, apex acuminate, base rounded, margin undulate, slightly erose at base, possessing numerous veins. *Filiform bracts* 8–15, up to 32 cm long, dark purple. *Flowers* narrowly triangular, buds dark red flowers greenish-purple when young, when older black-purple; perianth tube 5–6 mm long, white and purple inside. *Perianth lobes* 6, three outer lobes narrowly ovate or triangular, 10 × 3–5 mm, apex acute; three inner lobes broadly ovate to ovate-triangular, 8–10 × 4–6 mm, apex acuminate, recurved when flower fully opening. *Stamens* 6, attached to the base of perianth tube, beset with stigma lobes, dark purple to dark red, filament short, anther and connective forming a hood-like structure, individual stamens with 2 thecae, each theca 2 mm long. *Stigma* 3, ca. 5 mm in diameter, fleshy, apex 2-lobed with finely white pubescence, lobes slightly curved down; style 8–9 mm in diameter, sub-orbicular to round-triangular in outline, surface with minute black hairs. *Ovaries* inferior, obtriangular, 5–6 × 8–9 mm, dark red. *Fruit* berry, 3–4 × 1.5–2 cm, black to dark purple, triangular in transverse section; stalk 1.5–2 cm long. *Seeds* reniform to nearly half orbicular, 1.5–5 × 1–1.5 mm, slightly brown, 4–7-ribbed.

#### Other specimen examined.

VIETNAM. Khanh Hoa Province, Hon Ba Nature Reserve, growing under forest canopy, uncommonly along the riparian forest, alt. 400 m, 12°06.37'N, 108°59.44'E, 16 November 2017, *Truong Ba Vuong, Mang Van Lam, BV256a* (VNM!, the herbarium of Hon Ba Nature Reserve!).

#### Phenology.

Flowers were observed in April and May. Immature and mature fruits were observed in May and June.

#### Distribution and habitat.

*Taccakhanhhoaensis* is only known from Hon Ba Nature Reserve, Khanh Hoa Province, southern Vietnam. It was growing under the shade of the canopy on dry soil or close to the stream, where *Aporosatetragona* Tagane & V.S. Dang, *Bambusa* sp., *Barringtoniamacrostachya* (Jack) Kurz, *Calamus* sp., *Croton* sp., *Desmos* sp., *Goniothalamusflagellistylus* Tagane & V.S. Dang, *Ixora* sp., *Microcostomentosa* Sm., *Phyllanthusreticulatus* Poir. and *Streblusindicus* (Bureau) Corner are dominant.

#### Etymology.

The name of this species is derived from the name of the province Khanh Hoa, where the species was discovered.

#### Conservation status.

*Taccakhanhhoaensis* was collected from a small population under forest canopy in Hon Ba Nature Reserve at 300–400 m altitude. The forest habitat where we found this new species was frequently logged and disturbed. Therefore, *Taccakhanhhoaensis* is assessed as Critically Endangered (CR) based on the IUCN Red List Categories (IUCN 2012), although some individuals might be found by more thorough surveys.

#### Note.

*Taccakhanhhoaensis* is morphologically similar to *T.chantrieri* and *T.ampliplacenta*. The comparisons of morphological characters amongst these three species are summarised in Table 1.

**Table 1.** Morphological comparison of *Taccakhanhhoaensis* with its closest congeners (modified from Drenth 1972; Phengklai 1993; Ding and Larsen 2000; Pham 2000; Zhang et al. 2008; Baruah et al. 2015).

Characters	* T. khanhhoaensis *	* T. chantrieri *	* T. ampliplacenta *
Leaves			
– number	5 to 8	3 to 12	5 to 10
– shape of leaf lamina	oblong to obovate or oblong-elliptic	oblong to oblong-elliptic	oblong-obovate
– length of leaf lamina	30–45 cm	20–50(–60) cm	55 cm
– length of petiole	14–22 × 0.5–1 cm	11–43 × 0.2–0.5 cm	30–50 × 0.7–1.2 cm
Inflorescence			
– number of flowers	5 to 10	15 to 25	25
– length of scape	20–38 cm	6–63 cm	40–70 cm
– outer involucral bracts	ovate-lanceolate,	ovate-lanceolate,	lanceolate to oblong-ovate,
	4.5–5.5 × 2–2.5 cm	2–9 × 0.8–4 cm	6–8 × 3–4 cm
– inner involucral bracts	broadly ovate,	broadly ovate,	broad-triangular,
	6–6.5 × 4–5 cm	2.5–10 × 1.5–9 cm	10–16 × 8–10 cm
Flowers			
– colour of buds	dark red	green	black-purple
– outer perianth lobes	narrowly ovate or triangular,	oblong-ovate or narrowly triangular,	oblong,
	10 × 3–5 mm	5–12 × 3–8 mm	12 × 8 mm
– inner perianth lobes	broadly ovate,	broadly ovate or triangular,	broad-ovate,
	8–10 × 4–6 mm	4–11 × 4–12 mm	12 × 10 mm
Apex of stigma lobes	2-lobed	3-lobed	2-lobed
Ovaries	5–6 × 8–9 mm	2–7 × 3–5 mm	5–8 × 10 mm
Fruits	3–4 × 1.5–2 cm	2–4 × 1–2 cm	4–6 × 2–2.5 cm
Seeds	1.5–5 × 1–1.5 mm, slightly brown, 4–7-ribbed	2–4 × 1–2 mm, brown, 9–14-ribbed	2–3 × 1.5–2 mm, brownish-red, many ribbed

### Key to the species of *Tacca* in Vietnam

**Table:** 

1	Leaves lobed	**2**
–	Leaves entire	**3**
2	Leaves 3-lobed, each lobe pinnately. Filiform bracts present	*** T. leontopetaloides ***
–	Leaves 3–13-lobed, each lobe simple. Filiform bracts absent	*** T. palmata ***
3	Leaf-base attenuate and decurrent. Fruit dehiscent	*** T. plantaginea ***
–	Leaf-base attenuate but not decurrent. Fruit indehiscent	**4**
4	Inner two involucral bracts long petiolate	*** T. integrifolia ***
–	Inner two involucral bracts sessile	**5**
5	Inner involucral bracts suborbicular-fan-shaped	*** T. subflabellata ***
–	Inner involucral bracts broadly ovate	**6**
6	Inflorescence with 15–25-flowered. Apex of stigma lobes 3-lobed. Seeds reniform, brown, 9–14-ribbed	*** T. chantrieri ***
–	Inflorescence with 5–10-flowered. Apex of stigma lobes emarginated or 2-lobed. Seeds reniform, slightly brown, 4–7-ribbed	*** T. khanhhoaensis ***

## Supplementary Material

XML Treatment for Tacca khanhhoaensis
